# Distribution, Polymer Composition, and Exposure Risks of Microplastics in Bottled and Tap Water Distribution

**DOI:** 10.3390/molecules31132237

**Published:** 2026-06-25

**Authors:** Mariana Silva, Pedro Ideia, Carolina Pimenta-Fernandes, Ricardo Sousa, José S. Câmara, Rosa Perestrelo

**Affiliations:** 1 CQM—Centro de Química da Madeira, Universidade da Madeira, Campus da Penteada, 9020-105 Funchal, Portugal; 2028323@student.uma.pt (M.S.); pedro.di.freitas@madeira.gov.pt (P.I.); 2 Direção de Serviços de Monitorização, Estudos e Investigação do Mar, Direção Regional de Pescas (DSEIMar/DRP), Lota do Funchal, 1.° Piso, Rua Virgílio Teixeira, 9004-562 Funchal, Portugal; carolinapf115@gmail.com (C.P.-F.); ricardo.js.sousa@madeira.gov.pt (R.S.); 3 Observatório Oceânico da Madeira, Agência Regional para o Desenvolvimento da Investigação, Tecnologia e Inovação (OOM/ARDITI), Edifício Madeira Tecnopolo, Piso 0, Caminho da Penteada, 9020-105 Funchal, Portugal; 4 MARE—Marine and Environmental Sciences Centre, ARNET—Aquatic Research Network, Observatório Oceânico da Madeira, Agência Regional para o Desenvolvimento da Investigação, Tecnologia e Inovação (OOM/ARDITI), Edifício Madeira Tecnopolo, Piso 0, Caminho da Penteada, 9020-105 Funchal, Portugal; 5 Departamento de Química, Faculdade de Ciências Exatas e Engenharia, Universidade da Madeira, Campus da Penteada, 9020-105 Funchal, Portugal

**Keywords:** tap water, bottled water, microplastics, stereomicroscopy, µ-FTIR, EDI

## Abstract

Microplastic (MP) pollution in bottled and tap water poses escalating environmental and public health challenges due to MPs’ capacity to act as vectors for toxicants and pathogens. This study constitutes the first comprehensive evaluation of MPs in drinking water from Madeira Island, integrating detailed chemical and morphological characterisations alongside human exposure estimations. A total of 22 samples, comprising 10 bottled (four mineral, six flavoured) and 12 tap waters, were analysed via stereomicroscopy and micro-Fourier transform infrared (µ-FTIR) spectroscopy. Of the 428 particles detected, 65 were confirmed MPs, 223 were non-plastics, and 140 were indeterminate. Bottled waters were predominantly contaminated by polyethylene terephthalate (PET), polypropylene (PP), and polyethylene (PE), whereas tap waters exhibited a notable presence of PE, PP, polyester, and polyamide (PA). MPs predominantly measured under 400 µm and were transparent; fragments were the main form in bottled water, contrasting with fibres dominating tap waters. Concentrations ranged from 0.5 to 6 MPs/L, with flavoured waters exhibiting the highest average levels (2.00 ± 1.83 MPs/L), followed by tap (1.30 ± 0.80 MPs/L) and mineral waters (0.59 ± 0.37 MPs/L). Estimated daily intake (EDI) spanned 0.01–0.19 MPs/kg/day for adults and 0.05–0.68 MPs/kg/day for children, the latter exhibiting a 3.6-fold greater exposure. Although concentrations were lower than those in many global reports, the ubiquity of MPs underscores the critical need for standardised monitoring protocols, enhanced production standards, and rigorous risk assessments addressing chronic low-level human exposure, especially in insular environments.

## 1. Introduction

The growth in plastic production and consumption over the last few decades has resulted in ubiquitous environmental contamination, especially through the discharge of microplastics (MPs), plastic particles smaller than five mm, into the environment on land and in water [1,2,3]. By 2023, global plastic production had surpassed 400 million tons, and just 9.5% of that was produced from recycled material, with a large proportion entering waste streams that are resistant to degradation due to the intrinsic stability of most synthetic polymers [4]. Geyer et al. [5] reported that in 2015, roughly 9% of the world’s plastic waste was recycled, 12% was incinerated, and the remaining 79% was in landfills or illegally dumped.

MPs either result from the degradation of larger plastic waste, such as bottles, packaging and synthetic clothing (secondary MPs), or are intentionally manufactured at microscopic sizes for use in cosmetics, industrial abrasives, and packaging materials (primary MPs). Their widespread presence and persistence represent a significant and increasing threat to environmental integrity and public health [6,7].

Particularly concerning is the pathway of human exposure to MPs, which occurs through drinking water, including both bottled and tap water. Numerous studies have confirmed the presence of MPs in drinking water worldwide, with concentrations varying significantly depending on geographic location, water treatment processes, and sampling methods. Bottled water generally presents higher MP loads due to leaching from packaging materials and contamination during production, filling, and storage, with temperature fluctuations and extended shelf-life further accelerating polymer degradation and particle release [1,6,7,8,9,10,11,12,13]. The most prevalent polymers present are polyethylene terephthalate (PET), polypropylene (PP), polystyrene (PS), and polyethylene (PE), which are commonly used in drink containers, caps, seals, and filtering units [1,6,7,8,9,10,11,12,13]. MP contamination of drinking water is attributed to numerous vectors, such as atmospheric deposition, degradation of distribution systems (e.g., plastic pipe), wastewater effluent, and surface runoff [9,14,15].

The identification and quantification of MPs in water matrices are analytically challenging and require multi-step procedures involving sample concentration, removal of organic matter, and particle identification. Fourier Transform Infrared Spectroscopy (FTIR) [16,17], Raman spectroscopy [8,18], laser direct infrared spectroscopy (LD-IR) [1] and Scanning Electron Microscopy coupled with Energy-Dispersive X-ray Spectroscopy (SEM/EDX) [19] are the most employed methodologies for polymer identification. On the other hand, pyrolysis–gas chromatography–mass spectrometry (Py-GC-MS) has emerged as a complementary analytical approach for MP analysis, enabling sensitive polymer identification and bulk quantification through the thermal decomposition of plastic particles into characteristic pyrolysates, although it does not preserve particle-level information such as size, shape, or morphology [20,21]. Discrepancies in sampling protocols, particle size cut-offs, and analytical sensitivity, nevertheless, limit cross-study comparison and the development of standardised monitoring frameworks [11,22]. Further advancement toward standardisation has been achieved, but it is not yet completely realised, emphasising the need for further harmonisation of sampling and analysis techniques for global monitoring [3].

Ingestion of MPs via drinking water has raised severe public health concerns, since MPs have been found in human faeces, blood, placenta, lung, and hepatic tissue, indicating their systemic translocation and bioaccumulation capacity. Although the toxicokinetics of MPs in humans remain fully explored, in vitro and in vivo studies have indicated a range of adverse biological effects, including inflammatory responses, oxidative stress, cell membrane disruption, and endocrine dysregulation. Moreover, MPs also function as carriers of poisonous additives (e.g., bisphenol A, phthalates) and environmental contaminants (e.g., heavy metals, persistent organic pollutants), which increase their overall toxicological burden [1,2,23,24,25,26,27].

Despite mounting evidence, regulation remains limited and fragmented. The World Health Organization (WHO) [28] acknowledged MP presence in drinking water but refrained from proposing health-oriented guidelines due to insufficient toxicological data. The European Union (EU) [29], on the other hand, has adopted interim frameworks within the revised version of the Drinking Water Directive and proposed bans on intentionally added MPs under REACH [30]. A major regulatory advance was recently introduced through Commission Delegated Decision (EU) 2024/1441, which supplements Directive (EU) 2020/2184 by establishing a harmonised methodology for monitoring MPs in drinking water across Member States. This delegated decision defines the analytical framework for sampling, sample preparation, particle detection, and polymer identification and recommends a size range of 20–5000 µm for routine monitoring while also requiring the use of spectroscopic techniques for polymer confirmation. By introducing standardised quality control criteria and minimum performance requirements, this regulation represents a critical step toward harmonised and comparable microplastic monitoring in drinking water across the EU, directly addressing one of the main analytical limitations highlighted in the current literature [31]. In the meantime, countries like France, Canada, and the UK have begun to implement bans and monitoring programmes to prevent MPs’ contamination.

This study systematically evaluates the occurrence and characterisation of MPs in drinking water from Madeira Island, with detailed analysis of polymer composition, particle size, colour, and morphology using micro-Fourier Transform Infrared Spectroscopy (µ-FTIR). Additionally, human exposure assessments were conducted by calculating the Estimated Daily Intake (EDI) for both adults and children. To the best of our knowledge, this investigation represents the inaugural report on MPs’ presence in Madeira’s drinking water. By integrating robust analytical data with exposure modelling, the study enriches the existing body of knowledge on microplastic contamination and underscores the urgent need for targeted regulatory frameworks in insular environments.

## 2. Results and Discussion

Twenty-two drinking waters were analysed, including 10 bottled waters (four natural mineral and six flavoured/carbonated) and 12 tap waters, to detect MPs via stereomicroscopy and µ-FTIR. All detected particles were classified into three categories: MPs, non-plastics, and indeterminate. The indeterminate category comprised particles removed during stereomicroscopic examination or lost in drying, those with identification confidence below 70%, and opaque particles unsuitable for transmittance-based analysis.

In total, 428 particles were recorded: 65 MPs, 223 non-plastics, and 140 indeterminates. Bottled waters contained 26 MPs, 87 non-plastics, and 75 indeterminate particles, while tap waters included 39 MPs, 121 non-plastics, and 59 indeterminate particles. Procedural blanks yielded 17 particles (12 non-plastics, 5 indeterminate) with no MPs detected, confirming the absence of contamination. The absence of MPs in procedural blanks, combined with controlled laboratory conditions and polymer types consistent with known drinking water sources (e.g., PET, PE, PP), supports that the detected MPs originated from the analysed samples rather than external contamination. Chemical characterisation of the non-plastic fraction identified cellulose as the predominant material, accounting for 40% of all non-plastic particles (n = 90).

### 2.1. Qualification of MP in Drinking Water

**Figure 1:**
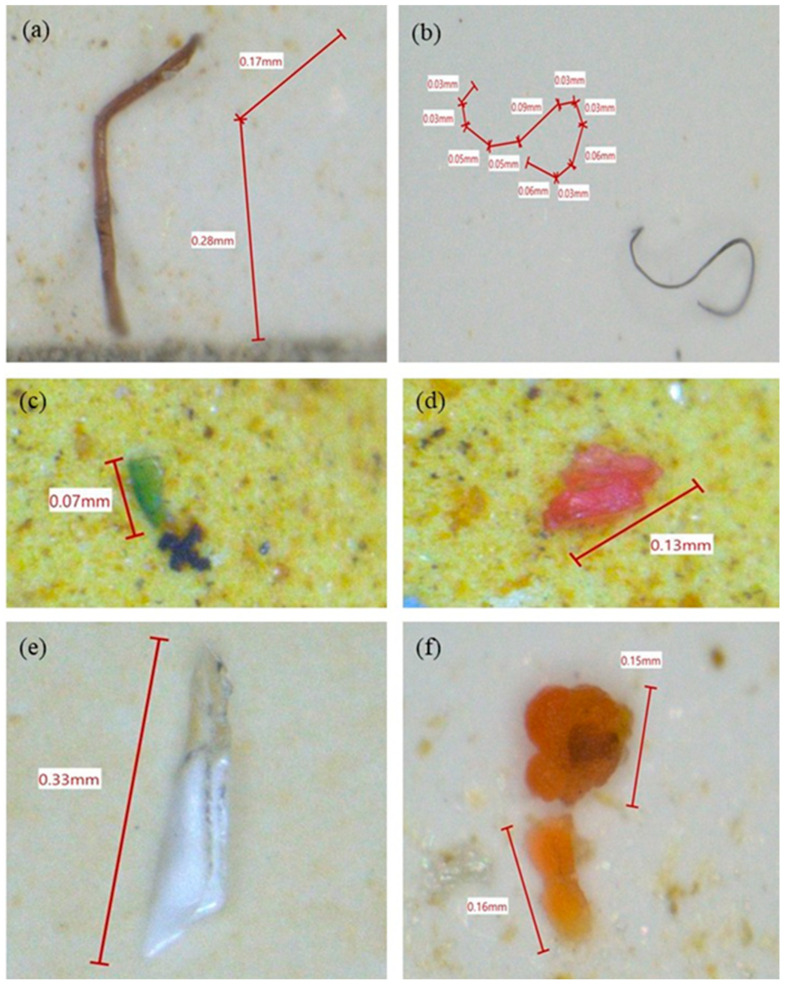
Representative microplastics in Madeira bottled and tap water: (**a**,**b**) fibres, (**c**–**f**) fragments.

The morphological assessment of MPs in drinking water from Madeira Island revealed two dominant shapes: fibres and fragments (Figure 1). The fragments consisted of irregularly shaped MPs encompassing films, foams, flakes, and foils. While these morphotypes are frequently classified separately in the literature, they were grouped in the present study owing to the inherent difficulties in their unambiguous discrimination.

**Figure 2:**
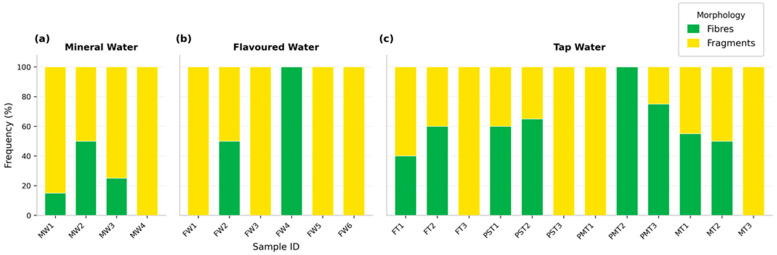
Distribution (%) of microplastic morphologies identified in the investigated water samples (**a**) Mineral water; (**b**) Flavoured water; (**c**) Tap water.

In mineral water, fragments were the dominant morphotype, averaging 77.8% of particles and ranging from 50% to 100% across samples, with fibres representing an average of 22.2%. Flavoured water showed a more variable profile, with some samples composed entirely of fragments (FW1, FW3, FW6) and others entirely of fibres (FW4, FW5), resulting in average proportions of 58.3% fragments and 41.7% fibres. Tap water exhibited the widest variability, including samples with exclusive fragments (PMT1, FT3) or fibres (PMT2, MT3) and several with balanced distributions (e.g., FT2, MT2), but overall presented a fibre-dominant pattern, with mean values of 52.4% fibres and 39.2% fragments (Figure 2). This distribution aligns with broader observations in the literature: one study found that bottled water contained around 65% fragments and 38% fibres [6,11], whereas tap water was overwhelmingly dominated by fibres (97%) within the size class larger than 100 µm [32]. Moreover, a recent UK-based analysis reported that fragments and fibres together comprised over 90% of MPs in both bottled and tap waters [11,12]. Fragments are likely generated from the breakdown of larger plastic items or abrasion of plastic fittings and pipelines in the water distribution network, whereas the fibres are often attributed to the release of microfibres from synthetic textiles during laundering, followed by airborne deposition during bottling, storage, or domestic handling [6]. While airborne deposition is recognised as a potential source of fibre contamination in MP studies, the strict contamination control measures applied in this work, together with the absence of MPs in procedural blanks, suggest that the observed fibres predominantly originated from the analysed water samples.

**Figure 3:**
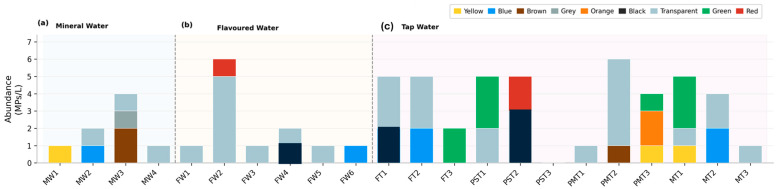
Microplastic colour profiles detected in bottled (**a**) Mineral water; (**b**) Flavoured Water; and (**c**) Tap water.

Across all drinking waters, transparent MP predominated, representing 75.0% in flavoured water, 64.3% in mineral water, and 56.4% in tap water (Figure 3). Mineral and flavoured waters exhibited low chromatic diversity, whereas tap water showed a broader colour spectrum, including red (7.7%) and green (7.7%) and other colours, such as grey, brown and orange in lower proportions (<3%). This prevalence of transparent MPs is consistent with previous literature, where transparent particles are often reported as dominant in drinking water, likely due to the degradation of clear PET bottles, polypropylene caps, or household synthetic fibres released during laundering [6,7]. The occasional presence of coloured particles (blue, brown, black, green, red, yellow) may be linked to specific sources such as dyed synthetic fabrics, pigmented plastic products, or paint residues. 

**Figure 4:**
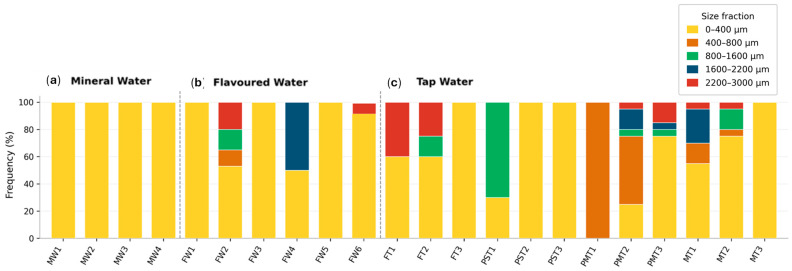
Length distribution (%) of microplastics observed in Madeira’s drinking waters: (**a**) Mineral Water; (**b**) Flavoured water; and (**c**) Tap water.

The size distribution of MPs in the bottled and tap waters revealed a predominance of smaller particles (Figure 4), particularly those in the <400 µm range, which accounted for 100% of particles in mineral water, 58.3% in flavoured water, and 40.9% in tap water. Larger size fractions were absent in mineral water but present in both flavoured and tap water: in flavoured water, particles of 400–800 µm and 800–1600 µm each represented 19.4 and 5.6%, respectively, while 1600–2200 µm and 2200–3000 µm each accounted for 8.3%. Tap water displayed the broadest size range, with 27.4% in the 400–800 µm fraction, 15.1% in the 800–1600 µm, 4.9% in the 1600–2200 µm, and 3.3% in the 2200–3000 µm.

These results align with previous studies on drinking water, where the <500 µm fraction is often the dominant one. Weisser et al. [33] and Sarlin et al. [8] reported that over 90% of MPs in mineral water were smaller than 500 µm. On the other hand, Shokunbi et al. [7] detected MPs in bottled water ranging from 200 to 2500 µm. Koelmans et al. [34] reported that smaller MPs are generally more abundant in drinking water, which is consistent with the predominance of smaller MPs observed in the present study. Differences in particle size distribution among mineral, tap, and flavoured waters may reflect differences in treatment, packaging, and handling processes. The lower occurrence of larger particles in mineral water may suggest more controlled filtration and bottling conditions. Environmental fragmentation mechanisms, such as UV photodegradation, mechanical wear, and hydrolysis, promote polymer chain scission and the progressive breakdown of larger plastics into smaller MP particles [35]. The absence of large particles in mineral water suggests stricter filtration or production control. In contrast, the wider size distribution of tap water may suggest heterogeneous contaminant pathways like household plumbing, environmental access, and sporadic release from residential use. The presence of high particle counts in flavoured water can be explained by additional steps taken during handling (e.g., flavouring, mixing, or packaging), where the filtration might not be as rigorous as that of mineral water.

**Figure 5:**
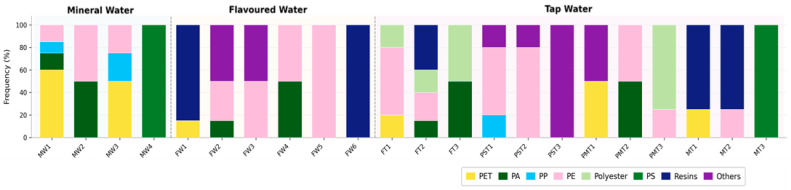
Frequency (%) of polymer types identified in all bottled and tap waters.

The polymer type distribution across mineral, flavoured, and tap waters revealed marked differences in both diversity and dominance patterns of polymers (Figure 5).

In mineral waters, PET, PP, and PE were the most prevalent, with PET ranging from 50% (MW3) to 57% (MW1), PP up to 14%, and PE up to 25%. These polymers are strongly associated with packaging sources: PET is the primary constituent of bottle bodies, PP is widely used for caps, and PE is often employed in liners and sealing rings. This composition mirrors numerous bottled-water surveys in which PET and PP dominate, often accompanied by PE, due to mechanical abrasion during capping, thermal stress during filling, and gradual wear during storage [11,36]. Interestingly, MW4 consisted entirely of PA, a polymer generally linked to synthetic fibres, suggesting contamination from processing lines, storage environments, or airborne fallout during bottling [37]. On the other hand, MW2 exhibited a more homogeneous polymer profile, consisting exclusively of PE (50%) and polyester (50%). This more restricted composition may indicate a narrower range of contamination sources and suggests a more uniform input of polymeric materials in this sample. The absence of PET and other polymers in MW2 further highlights the variability observed among mineral waters, reinforcing the influence of source-specific factors such as packaging material, bottling conditions, or storage-related contamination.

Flavoured waters displayed higher heterogeneity, with several samples exhibiting single-polymer dominance: PET (100%) in FW1, PVC (100%) in FW3, PE (100%) in FW6, and “resins” (100%) in FW5. Mixed profiles included PET (17%), PE (33%) and polyester (33%) in FW2, and PE (50%) and others (50%) in FW4. This variability may reflect brand-specific manufacturing processes, use of different closure or liner materials, and the greater complexity of flavoured-water production lines (e.g., syrup dosing equipment), which may introduce additional polymer types. Tap waters were characterised primarily by PE and PP, often in combination with polyester, PET or PA. For example, PST1 contained PE (60%) and PP (20%), with “resins” accounting for the remaining 20%, while PMT2 comprised equal proportions of PET and polyester (50% each). This pattern is consistent with large-scale tap-water surveys, in which PE and PP dominate, followed by polyester and PA, reflecting polymeric components in distribution networks, treatment plants, and possible ingress from domestic plumbing [13,33]. Polyester and PA are commonly found as fibres, originating from laundering, textile degradation, or airborne deposition in treatment facilities and laboratories [33,37]. Occasional detections of PVC, PS, PE, and “resins” in tap waters also align with literature, as PVC piping and PE connectors are widely used in municipal systems, while PS can derive from disposable labware or food-contact foams [38].

Inclusive, the polymer profiles observed here align well with published data for drinking waters: PET, PP, and PE as primary constituents in bottled waters; PE, PP, and polyester/PA dominance in tap waters; and sporadic detection of less common polymers such as PVC, PS, PE, and PTFE [11,13,33]. The notable variation among flavoured waters, including single-polymer dominance, likely reflects differences in materials and equipment between production lines, and may warrant targeted investigation. The unexpected abundance of PTFE in certain samples highlights the importance of thorough contamination control and transparent reporting of polymer identification thresholds, spectral quality, and blank corrections to ensure robust interpretation.

### 2.2. Quantification of MP in Drinking Water

**Figure 6:**
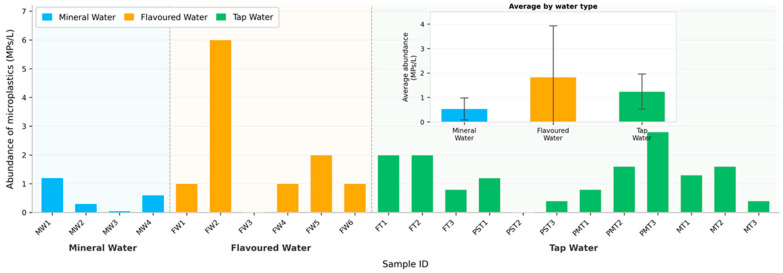
Microplastic abundance (MP/L) quantified in Madeira bottled and tap waters.

This study provides the first assessment of MP abundance in different types of drinking water—mineral, flavoured, and tap—collected across the Madeira Island region. A summary of the main findings is presented in Figure 6.

The results contribute to the expanding body of evidence indicating the presence of MPs in human drinking water supplies. Quantification analysis demonstrated that FW2 (flavoured bottled water) had the highest number of MPs (six MPs/L), while no MPs were recorded in PST3 (tap water). All reported MP concentrations were above the MDL (0.5 MPs/L), confirming that the quantified values represent measurable contamination levels rather than background noise. Although statistical analysis (one-way ANOVA, *p* = 0.208) did not reveal significant differences between water types, the mean MP concentrations showed a clear trend: flavoured water (2.00 ± 1.83 MPs/L) > tap water (1.30 ± 0.80 MPs/L) > mineral water (0.59 ± 0.37 MPs/L). Pairwise comparisons using Welch’s *t*-tests confirmed the absence of statistically significant differences; however, effect size analysis (Cohen’s *d*) suggested substantial differences, particularly between mineral and flavoured water (*d* = −0.88, large effect), and mineral and tap water (*d* = −0.75, medium-to-large effect). It is noteworthy that the concentration of MPs in the current study was lower than reported in studies conducted in other countries [1,7,8]. A possible explanation for elevated MP abundance in commercial flavoured water may be linked to extensive processing and packaging steps, which could contribute to introducing MPs through mechanical abrasion or leaching from plastic components such as bottle caps, multilayered linings, or labelling adhesives. These observations are consistent with previous studies reporting increased MP load in carbonated or flavoured beverages compared to mineral water [39,40].

On the other hand, tap water was not subject to industrial packaging, maybe the accumulation of MPs in these types of drinking water could be explained by the MPs through ageing infrastructure (e.g., plastic pipes, tanks) or inefficient treatment protocols. Tap water treatment plants are typically designed for decontamination by chemicals and microorganisms but not micrometric particle removal [41]. This could be the explanation for the presence of intermediate levels of contamination in tap water samples of various municipalities of Madeira. It is noteworthy that no MPs were detected in procedural blank samples, supporting the effectiveness of the contamination control procedures implemented during sample processing and analysis. This supports that the reported MP abundances are primarily derived from the analysed samples rather than from procedural artefacts [42]. Despite the limited sample size restricting the broader generalizability of these findings, the consistently higher MP abundance observed in flavoured water warrants attention due to the potential for repeated dietary exposure through regular consumption. While. Although MPs’ health impacts remain under investigation, repeated exposure may pose toxicological concerns, considering their ability to act as carriers of plastic-associated additives and adsorbed environmental contaminants [43,44].

### 2.3. Estimated Daily Intake (EDI) of Microplastics

The EDI of MPs, calculated using ingestion rates of 2.2 L/day for adults (70 kg bw) and 1.8 L/day for children (16 kg bw), revealed marked differences between water types (Table 1).

On average, flavoured water exhibited the highest particle concentration (2.00 MPs/L; median = 1.00; range = 1.00–6.00), resulting in mean EDIs of 0.06 MPs/kg/day for adults and 0.23 MPs/kg/day for children. Tap water showed intermediate values (mean = 1.30 MPs/L; median = 1.40; range = 0.00–2.80), corresponding to EDIs of 0.04 and 0.15 MPs/kg/day for adults and children, respectively. Mineral water presented the lowest concentrations (mean = 0.59 MPs/L; median = 0.50; range = 0.20–1.17), with EDIs of 0.02 (adults) and 0.07 MPs/kg/day (children). Across all water types, children’s EDIs were approximately 3.6 times higher than those of adults due to lower body weight and relatively high-water consumption. Notably, extreme values in flavoured (FW2 = 6.00 MPs/L) and tap water (MT1 = 2.80 MPs/L) substantially influenced mean values, highlighting the importance of reporting both mean and median concentrations. Previous studies have reported EDI for MPs from drinking water ranging from approximately 0.31 to 13 MPs/kg/day in adults, with infants showing comparatively higher exposure levels up to ~5 MPs/kg/day. Broader dietary exposure estimates, particularly when bottled water and additional food sources are considered, range from several hundred to more than one million particles per person per day [1,7]. In contrast, our calculated EDIs (0.01–0.19 MPs/kg/day for adults and 0.05–0.68 MPs/kg/day for children) are substantially lower, reflecting the relatively low MP particle concentrations observed in our sampled waters and reinforcing the value of reporting both mean and median values to account for extremes. Nevertheless, our calculated EDIs are quite higher than those reported by Makhdoumi et al. [10], who reported EDIs 0.01–0.02 MPs/kg/day for adults and 0.05–0.07 MPs/kg/day for children in bottled mineral waters. It should be noted that these exposure estimates are based exclusively on µ-FTIR-confirmed MPs above the established MDL, ensuring conservative and analytically robust exposure assessment.

## 3. Materials and Methods

### 3.1. Sample Collection

As part of the sampling design, four commercially available brands of mineral water (Code: MW1–MW4) and six brands of flavoured and/or carbonated water (FW1–FW6) were purchased in supermarkets on Madeira Island. All products were packaged in single-use polyethylene terephthalate (PET) bottles, representative of the formats most frequently consumed by the general population (Table S1). To preserve brand confidentiality, each bottle was assigned a unique alphanumeric code. Immediately after acquisition, all samples were transported to the laboratory under controlled conditions and stored at 4 °C until analysis.

In parallel, tap waters were collected from four municipalities on Madeira Island—Funchal (FT1–FT3), Machico (MT1–MT3), Porto Moniz (PMT1–PMT3), and Porto Santo (PST1–PST3), selected based on population density and/or geographic dispersion to ensure spatial representativeness across the region. In each municipality, three civil localities were chosen according to their demographic or spatial relevance, and a single sampling site was defined in each locality (Figure S1). From each sampling site, 2 L of tap water were collected in pre-cleaned, inert glass containers using standard protocols to avoid cross-contamination and ensure sample integrity. All tap waters were transported under refrigeration (4 °C) and stored under identical conditions until further MP analyses.

Each bottled water brand and tap water sampling point was treated as an independent environmental sample (n = 1). For each sample, a single 2 L aliquot was collected and processed for MP analysis. Thus, the reported dataset reflects one analytical determination per sampling unit, consistent with the exploratory monitoring design adopted in this study.

### 3.2. Quality Assurance and Contamination Control

To minimise the risk of MP cross-contamination during sample processing, all procedures were carried out under a laminar flow hood. Researchers wore nitrile gloves and cotton laboratory coats throughout all experimental stages to reduce the introduction of synthetic fibres. Before analysis, all sample containers were rinsed with 96% ethanol to eliminate potential surface contaminants and then filtered through 0.45 µm filters using distilled water to prevent airborne contamination. Laboratory equipment was thoroughly washed with distilled water after each use and subsequently wrapped in aluminium foil to prevent airborne contamination. To monitor background contamination, procedural blanks consisting of filtered deionised water were processed in parallel with samples under identical conditions (n = 3). These blanks underwent the same filtration, drying, and analytical procedures. Procedural blanks yielded a total of 17 particles, all of which were classified as non-plastic (n = 12) or indeterminate (n = 5), with no MPs detected. This indicates negligible background MP contamination under the applied laboratory conditions. Therefore, no blank correction was applied to MP counts. Air exposure time during filtration and sample handling was minimised, and all filtration apparatus were covered with watch glasses to further reduce the risk of airborne particle deposition. Analytical replication at the level of sample filtration was not performed due to the exploratory nature of the study and the high analytical burden associated with manual particle isolation and µ-FTIR confirmation. To ensure data reliability, all samples were processed under strictly standardised conditions, and procedural blanks (n = 3) were analysed in parallel under identical conditions to assess background contamination and methodological consistency.

### 3.3. Filtration and Characterisation of Microplastics

Each sample was filtered under reduced pressure using a Gardner Denver Welch vacuum pump (model 412021; Gardner Denver Thomas Inc., Mount Prospect, IL, USA). Filtration was performed using individually packaged, sterile Whatman^®^ filters (0.45 µm pore size; 47 mm diameter) mounted on a vacuum filtration unit. A watch glass was placed over the apparatus to reduce the risk of airborne particle contamination. Post-filtration, filters were handled with sterile tweezers and transferred to clean Petri dishes, then dried in a ventilated oven at 45 °C for at least 24 h. Dried filters were stored in aluminium foil-covered trays, clearly labelled, and kept in a dark, dust-free environment until further analysis.

MPs were screened and classified based on shape, colour, and size using a stereomicroscope. The particle size classes (<400 µm, 400–800 µm, 800–1600 µm, 1600–2200 µm, and 2200–3000 µm) were established based on the effective operational range of the analytical workflow, considering the combined constraints of membrane filtration, stereomicroscopic visual detection, manual particle handling, and subsequent µ-FTIR confirmation, rather than representing universal or regulatory size categories. According to established classification criteria [45], MPs were categorised as fibres or fragments. The visual inspection was supported by image acquisition using a Leica Stereoscope (model Sapo; Wetzlar, Germany), coupled with a Leica Flexcam C5 camera, running Leica Enersight software, version 1.0.1 (Germany), and all observed particles were measured, documented and coded. Suspected MPs were systematically collected for further analysis. To minimise false positives, only particles meeting established visual criteria for MPs (e.g., absence of cellular structures, homogeneous colour, and resistance to fragmentation under manipulation) were selected for further analysis.

The chemical MP identification was done using a Spotlight 200i µ-FTIR microscope coupled with a Spectrum Two FT-IR spectrometer, version Spectrum 10.5.2 (PerkinElmer, Waltham, MA, USA), following an established internal protocol. The particles were deposited onto gold mirror substrates, which served as reflective backgrounds to enhance spectral quality and minimise interference. The system first performed an automated phototile scan across the full surface of the gold mirror, generating a high-resolution composite image of the scanned area. Based on this image, each detected MP was manually marked using 2 to 3 region-specific markers, with the marker area adjusted according to the particle’s dimensions. This allowed for precise positioning of the infrared beam for optimal spectral acquisition. The µ-FTIR analysis was conducted in reflectance mode, with the following optimised instrumental parameters: spectral range of 4000–700 cm^−1^, spectral resolution of 4 cm^−1^, and 32 scans per particle. For selected samples requiring enhanced signal-to-noise ratios, 64 scans were performed. All spectra were acquired under controlled laboratory conditions to ensure reproducibility and accuracy. Spectral data were processed using Spectrum software version 10.5.2 (PerkinElmer), and chemical identification of the MPs was conducted by comparison against an integrated polymer reference spectral library. The degree of match between the sample spectrum and the reference spectra was expressed as a percentage of similarity, and only particles with a match of ≥70% were considered positively identified. Particles below this threshold were categorised as indeterminate and excluded from MP quantification.

### 3.4. Method Detection Limits for Microplastic Quantitation

The method detection limit of the procedure was assessed in two ways, namely the limit of particle detection and the limit of concentration detection.

The minimum possible size that could be detected using the procedure depended on the resolution capabilities of the stereomicroscope and µ-FTIR instrument, as well as the pore size of the filtration membrane (0.45 µm). In practice, reliable identification was achieved for particles ≥10–20 µm due to instrumental limitations.

The method detection limit (MDL) used for MP quantitation was determined based on blank analysis and was calculated using the formula: MDL = mean of blank + 3 × standard deviation. As there were no MPs detected in any procedural blanks, the MDL was set conservatively at 1 MPs/sample, or 0.5 MPs/L based on 2 L filtration volumes. MP concentrations below this value were considered non-quantifiable.

### 3.5. Estimated Daily Intake (EDI)

The EDI of MPs through drinking water consumption was calculated to assess potential human exposure, using the equation:

$$\mathrm{EDI}\;(\mathrm{MPs}/\mathrm{kg}/\mathrm{day})\;=\;\frac{C\times IR}{bw}$$

where C represents the concentration of MPs in water samples (particles/L), IR is the ingestion rate of drinking water (L/day), and bw is the body weight (kg) of the consumer, 70 kg for adults and 16 kg for children. The IR was assumed to be 2.2 L/day for adults and 1.8 L/day for children, based on average daily bottled water consumption data in high-exposure scenarios. This method has been previously applied in similar exposure assessment studies across various countries [7,8,10].

### 3.6. Statistical Analyses

Statistical analysis was performed to evaluate differences in MP concentration among mineral, flavoured, and tap waters. Descriptive statistics, including mean, standard deviation (SD), median, and range, were used to summarise MP concentrations (MPs/L) and estimated daily intake (EDI) values. Differences in MP concentration among water types were assessed by one-way analysis of variance (ANOVA), followed by Welch’s *t*-tests for pairwise comparisons. Effect sizes were estimated using Cohen’s d. Statistical significance was set at *p* < 0.05 using IBM SPSS 29.0 software (IBM Corp, Armonk, NY, USA).

## 4. Conclusions

This study establishes the first baseline quantification of MP contamination in both bottled and tap drinking water from Madeira Island, revealing distinct variations in polymer composition, morphology, and size distribution between water sources. Although measured MP concentrations were comparatively lower than most global reports, their ubiquitous presence, particularly elevated in flavoured bottled waters, indicates ongoing contamination sources. The dominance of PET, PP, and PE in bottled waters reflects packaging-related contributions, whereas the detection of PE, PP, polyester, and PA in tap water suggests contamination arising from household plumbing and water distribution infrastructure. The EDI values highlight a significantly higher exposure in children, raising concerns over potential health effects given MPs’ capacity to adsorb environmental pollutants and toxic additives. A limitation of this study is the absence of replicate sampling and filtration for each water source, which restricts the assessment of intra-sample variability. Nevertheless, the study was designed as an exploratory baseline survey aimed at establishing the first occurrence data for MPs in Madeira’s drinking water. These findings underscore an urgent need for standardised analytical methodologies, implementation of targeted mitigation strategies, and continuous surveillance, especially in insular regions where water supply chains may be particularly susceptible to contamination.

## Figures and Tables

**Table 1:** Estimated daily intake (MPs/kg/day) for both adults and children.

Samples	Code	Concentration (MPs/L)	EDI (MPs/kg/day)	
			Adult	Children
Mineral Water	MW1	1.17	0.04	0.13
	MW2	0.33	0.01	0.04
	MW3	0.67	0.02	0.08
	MW4	0.20	0.01	0.02
Flavoured Water	FW1	1.00	0.03	0.11
	FW2	6.00	0.19	0.68
	FW3	1.00	0.03	0.11
	FW4	2.00	0.06	0.23
	FW5	1.00	0.03	0.11
	FW6	1.00	0.03	0.11
Tap Water	FT1	2.00	0.06	0.23
	FT2	2.00	0.06	0.23
	FT3	0.80	0.03	0.09
	PST1	2.00	0.06	0.23
	PST2	1.20	0.04	0.14
	PST3	-	-	-
	PMT1	0.40	0.01	0.05
	PMT2	0.80	0.03	0.09
	PMT3	1.60	0.05	0.18
	MT1	2.80	0.09	0.32
	MT2	1.60	0.05	0.18
	MT3	0.40	0.01	0.05

## Data Availability

The original contributions of this study are fully included in the article. Any additional inquiries should be addressed to the corresponding authors.

## Supplementary Materials

The following supporting information can be downloaded at: https://www.mdpi.com/article/10.3390/molecules31132237/s1, Figure S1: Geographically and demographically representative tap water sampling sites were strategically selected from the municipalities of Funchal, Machico, Porto Moniz, and Porto Santo on Madeira Island to capture spatial variability across the island’s diverse urban and rural populations; Table S1: Sample information.

## Abbreviations

The following abbreviations are used in this manuscript:

µ-FTIR	micro-Fourier transform infrared spectroscopy
EDI	Estimated daily intake
LD-IR	laser direct infrared spectroscopy
MPs	microplastics
PA	polyamide
PE	polyethylene
PET	polyethylene terephthalate
PP	polypropylene
PS	polystyrene
PVC	polyvinyl chloride
SEM/EDX	Scanning Electron Microscopy coupled with Energy-Dispersive X-ray Spectroscopy
