# Supplementary material for: Distribution, Polymer Composition, and Exposure Risks of Microplastics in Bottled and Tap Water Distribution
Source: Molecules. 2026 Jun 25;31(13):2237. doi: 10.3390/molecules31132237 (PMC13363690; doi:10.3390/molecules31132237)
Supplement: Supplementary file 1 [file molecules-31-02237-s001.zip › molecules-4280467-supplementary.pdf]

Distribution, Polymer Composition, and Exposure Risks of Microplastics in Bottled and Tap Water

Mariana Silva [1], Pedro Ideia [1,2], Carolina Pimenta-Fernandes [2], Ricardo Sousa [2,3,4], José S. Câmara[1,5*], Rosa Perestrelo [1,*]

[1] CQM – Centro de Química da Madeira, Universidade da Madeira, Campus da Penteada, 9020-105 Funchal, Portugal

[2] Direção de Serviços de Monitorização, Estudos e Investigação do Mar, Direção Regional de Pescas (DSEIMar/DRP), Lota do Funchal, 1.º Piso, Rua Virgílio Teixeira, 9004-562 Funchal, Portugal

[3] Observatório Oceânico da Madeira, Agência Regional para o Desenvolvimento da Investigação, Tecnologia e Inovação (OOM/ARDITI), Edifício Madeira Tecnopolo, Piso 0, Caminho da Penteada, 9020-105 Funchal, Portugal

[4] MARE – Marine and Environmental Sciences Centre / ARNET – Aquatic Research Network, Agência Regional para o Desenvolvimento da Investigação, Tecnologia e Inovação (OOM/ARDITI), Edifício Madeira Tecnopolo, Piso 0, Caminho da Penteada, 9020-105 Funchal, Portugal

[5] Departamento de Química, Faculdade de Ciências Exatas e Engenharia, Universidade da Madeira, Campus da Penteada, 9020-105 Funchal, Portugal

*Corresponding author. Tel: +351 291705119; Fax: +351 291705149

E-mail address: jsc@staff.uma.pt (José S. Câmara); rmp@staff.uma.pt (Rosa Perestrelo)

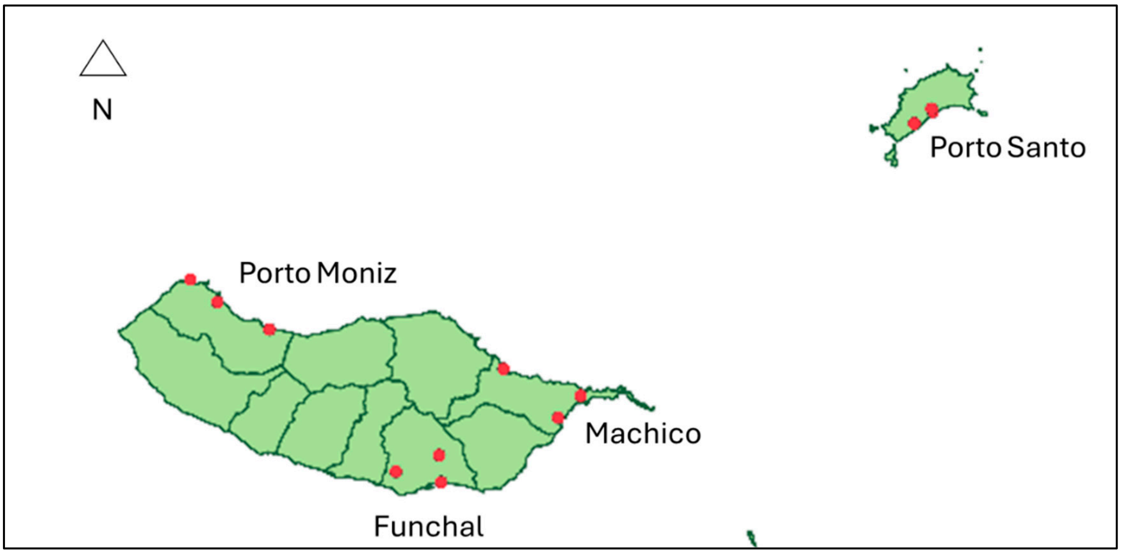

Figure S1. Geographically and demographically representative tap water sampling sites were strategically selected from the municipalities of Funchal, Machico, Porto Moniz, and Porto Santo on Madeira Island to capture spatial variability across the island's diverse urban and rural populations.

Table S1. Sample information

	CODE	Samples	Volume [illegible]
Commercial Water	MW1	Mineral water	6
	MW2	Mineral water	6
	MW3	Mineral water	6
	MW4	Mineral water	5
	FW1	Flavour/carbonated water	2
	FW2	Flavour/carbonated water	2
	FW3	Flavour/carbonated water	2
	FW4	Flavour/carbonated water	2
	FW5	Flavour/carbonated water	2
	FW6	Flavour/carbonated water	2
Tap Water	FT1	Funchal (Monte)	2
	FT2	Funchal (Santa Maria Maior)	2
	FT3	Funchal (Santo António)	2
	PST1	Porto Santo	2
	PST2	Porto Santo	2
	PST3	Porto Santo	2
	PMT1	Porto Moniz (Ribeira da [illegible]	2
	PMT2	Porto Moniz (Seixal)	2
	PMT3	Porto Moniz	2
	MT1	Machico (Porto da Cruz)	2
	MT2	Machico (Caniçal)	2
	MT3	Machico	2
Control	CW1	Desionize water	2
	CW2	Desionize water	2
	CW3	Desionize water	2
